# Alterations of Cytochrome P450s and UDP-Glucuronosyltransferases in Brain Under Diseases and Their Clinical Significances

**DOI:** 10.3389/fphar.2021.650027

**Published:** 2021-04-21

**Authors:** Yun Sheng, Hanyu Yang, Tong Wu, Liang Zhu, Li Liu, Xiaodong Liu

**Affiliations:** Center of Pharmacokinetics and Metabolism, School of Pharmacy, China Pharmaceutical University, Nanjing, China

**Keywords:** brain, cytochrome P450s, UDP- glucuronosyltransferases, endogenous substances, drug response, diseases

## Abstract

Cytochrome P450s (CYPs) and UDP-glucuronosyltransferases (UGTs) are both greatly important metabolic enzymes in various tissues, including brain. Although expressions of brain CYPs and UGTs and their contributions to drug disposition are much less than liver, both CYPs and UGTs also mediate metabolism of endogenous substances including dopamine and serotonin as well as some drugs such as morphine in brain, demonstrating their important roles in maintenance of brain homeostasis or pharmacological activity of drugs. Some diseases such as epilepsy, Parkinson’s disease and Alzheimer’s disease are often associated with the alterations of CYPs and UGTs in brain, which may be involved in processes of these diseases via disturbing metabolism of endogenous substances or resisting drugs. This article reviewed the alterations of CYPs and UGTs in brain, the effects on endogenous substances and drugs and their clinical significances. Understanding the roles of CYPs and UGTs in brain provides some new strategies for the treatment of central nervous system diseases.

## Introduction

Cytochrome P450s (CYPs, also known as CYP450s) and UDP-glucuronosyltransferases (UGTs) are expressed in brain, showing certain physiological roles. CYPs, such as CYP3A, CYP2D, CYP2E, CYP2B, CYP1A, CYP46A1 and CYP2C, are mainly distributed in neurons and astrocytes (McMillan and Tyndale, 2018), while UGTs such as UGT1A and UGT2B are mainly expressed in astrocytes and endothelial cells (Ouzzine et al., 2014). CYPs and UGTs expressed in brain mediate the synthesis and metabolism of some endogenous substances and central nervous system (CNS) drugs in brain. These enzymes, along with blood-brain-barrier (BBB), are involved in homeostasis of endogenous substances in brain. Although compared with them in liver, the total amount of metabolic enzymes in brain is limited, which are unlikely to affect the systemic drug metabolism, they play an important role in CNS drug metabolism and response.

More importantly, the characteristics of brain CYPs and UGTs are often different from liver. For example, growth hormone receptor knockout significantly lowered brain *Cyp2d* mRNA levels without affecting expressions of hepatic *Cyp2d* in mice (Zhang et al., 2018). The CYP2D activity and protein levels increased in the hippocampus, hypothalamus and striatum of rats with aging, but decreased in the frontal cortex and liver of senescent rats (Haduch et al., 2020b). Nicotine induced CYP2B mRNA and protein levels in rat brain but not in liver (Garcia et al., 2017). As well, *UGT1A3* mRNA was up-regulated by nicotine in humanized *UGT1* mouse brain but not in liver (Sakamoto et al., 2015). These dissimilarities may be attributed to different distribution of nuclear receptors involved in the regulation of CYPs and UGTs expressions in liver and brain (Nishimura et al., 2004).

It is generally accepted that brain CYPs and UGTs mediate metabolism of endogenous substances (such as neurotransmitters and neurosteroids) and therapeutic drugs on CNS (such as antiepileptics and analgesics). Some CNS diseases also affect expressions and functions of brain CYPs and UGTs, leading to disorder of endogenous substances and altering drug disposition or pharmacological activities/toxicities in brain. The roles of brain CYPs in the progression of several CNS diseases were comprehensively presented in some reviews (Haduch and Daniel, 2018; Navarro-Mabarak et al., 2018). Here, we introduced roles of brain CYPs and UGTs in metabolism of several endogenous substances (dopamine, serotonin, bilirubin, arachidonic acid, cholesterol and allopregnanolone) and CNS drugs (antiepileptics, analgesics and antipsychotics), and then focused on the alterations in expressions and functions of brain CYPs and UGTs under some diseases (epilepsy, Parkinson’s disease, Alzheimer’s disease, depressive disorder, smoking and alcoholism) as well as their impacts on homeostasis of endogenous substances and drug efficacy, which would provide some new ideas and strategies for understanding CNS disease progression and rational drug use.

## The Roles of Cytochrome P450s and UDP-Glucuronosyltransferases in Endogenous Substances

Several reports have revealed that CYPs and UGTs in brain are involved in metabolism of some endogenous substances, such as dopamine, serotonin, bilirubin, arachidonic acid, cholesterol and allopregnanolone, whose significances in homeostasis of brain are greater than CYPs and UGTs in liver.

### Dopamine

**FIGURE 1:**
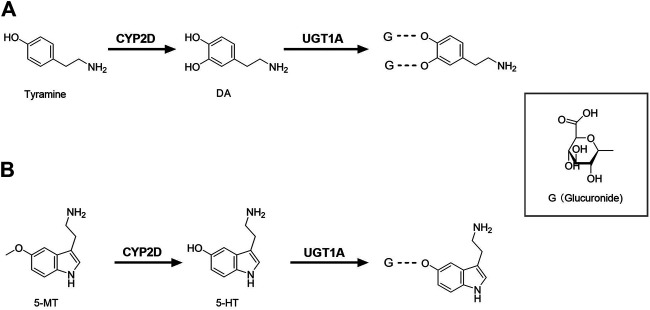
Brain CYP2D and UGT1A mediate the metabolism of DA **(A)** and 5-HT **(B)**. CYP2D, Cytochrome P450 2D; DA, dopamine; G, glucuronide; UGT1A, UDP-glucuronosyltransferase 1A; 5-HT, serotonin; 5-MT, 5-methoxytryptamine.

In the classical synthesis pathway, phenylalanine is converted to tyrosine by phenylalanine hydroxylase, then oxidized to levodopa (L-dopa) by tyrosine hydroxylase which is the rate-limiting step, and finally further metabolized to dopamine (DA) by aromatic amino acid decarboxylase. DA can also be formed from tyramine by brain CYP2D. Rat brain microsomes metabolized tyramine into DA, which was inhibited by both CYP2D inhibitor quinine and anti-CYP2D4 antibodies (Bromek et al., 2010), indicating that CYP2D4 is the regulatory subtype of tyramine hydroxylated to DA in rat brain (rat CYP2D4 is a potential homolog of human CYP2D6) (Figure 1A). It was reported that the specific CYP2D inhibitor quinine, given to reserpinized rats treated with α-methyl-p-tyrosine (a blockade of the classical pathway of DA synthesis from tyrosine) and pargyline (an inhibitor of monoamine oxidase A and B), significantly decreased DA levels in the striatum and nucleus accumbens by 35 and 26%. Microdialysis analysis further demonstrated that tyramine (100 μM), given locally via microdialysis probe, significantly increased contents of DA in the striatum of reserpinized rats with α-methyl-p-tyrosine, which was obviously attenuated by quinine (Bromek et al., 2011). Human CYP2D6 was more efficient than rat CYP2D4 (Bromek et al., 2010), suggesting that the alternative pathway of DA synthesis via CYP2D is more important in the human brain than in the rodent brain. Tyramine does not cross BBB and brain tyramine mainly comes from the aromatic hydroxylation of phenylethylamine or tyrosine decarboxylation (Hiroi et al., 1998; Berry, 2004). The basal levels of m-tyramine and p-tyramine in rat striatum were respectively 18.2 and 80.2 nM (Dougan et al., 1983). Rat brain microsomes were reported to catalyze the hydroxylation of m-tyramine and p-tyramine to DA, but m-tyramine (Km = 954 μM; Vmax = 0.94 pmol/mg protein/min and CL = Vmax/Km = 0.00098 μL/mg protein/min) was more efficiently metabolized than p-tyramine (Km = 1,303 μM; Vmax = 0.54 pmol/mg protein/min, CL = 0.0004 μL/mg protein/min) (Bromek et al., 2010). The estimated total clearance of DA formation from tyramine CLtotal = 0.0014 μL/mg protein/min, which were greatly less than clearance of L-dopa formation from L-tyrosine (Km = 79 μM; Vmax = 66 pmol/mg protein/min, CL = 0.83 μL/mg protein/min) in preoptic homogenates of rats (Perez et al., 1987), inferring minor roles of CYP2D-mediated DA synthesis under normal condition. However, the significance of CYP2D-mediated DA synthesis in the human brain may increase under specific conditions, such as blockade of classical synthesis pathway or pathological deficiency (i.e. inhibition of tyrosine hydroxylase or aromatic amino acid decarboxylase) (Goswami et al., 2017; Salvatore et al., 2019) or induction of brain CYP2D (Mann et al., 2008; Haduch et al., 2018).

DA is mainly metabolized to dihydroxyphenyl acetic acid (DOPAC) by monoamine oxidase (MAO) and aldehyde dehydrogenase, and DOPAC can be metabolized by catechol methyltransferase (COMT) to homovanillic acid (HVA). Although the main routes of DA metabolism have been established, there are still many unknown metabolites in various brain regions. It was reported that inhibition of CYP2E1 increased the extracellular DA in the substantia nigra (Nissbrandt et al., 2001) and significantly altered metabolite pattern of L-dopa (Niazi Shahabi et al., 2003), indicating that CYP2E1 might participate in DA metabolism or release. Glucuronide and sulfate conjugates of DA were detected in microdialysis samples of rat brain (Uutela et al., 2009). The amounts of DA glucuronide in rat cerebrospinal fluid (CSF) were higher than that of DA sulfate (Wang et al., 1983) and the mRNA expressions of *UGT1A1*, *UGT1A3*, *UGT1A6* and *UGT1A10* were also detected in the human brain (Kutsuno et al., 2015), suggesting that glucuronidation also serves as a pathway of DA metabolism in brain (Figure 1A). However, among the tested 19 human recombinant UGTs, only UGT1A10 had a relatively high affinity (Itäaho et al., 2009). It was reported that DOPAC and HVA are still the main metabolites of DA in human brain microdialysates (Suominen et al., 2013), indicating that the glucuronide conjugate of DA seem to be minor in human. DA glucuronidation and its physiological significance in brain need to be further explored.

### Serotonin

Tryptophan is firstly catalyzed by tryptophan hydroxylase to 5-hydroxytryptophan, and then catalyzed by 5-hydroxytryptophan decarboxylase to serotonin (5-HT). Data from recombinase and transgenic mice confirmed that the human CYP2D6 subtype catalyzed 5-methoxytryptamine (5-MT) to 5-HT in liver (Yu et al., 2003), which was also confirmed by rat brain CYP2D *in vitro* and *in vivo* (Haduch et al., 2013, 2015). The higher abundance of 5-HT and 5-hydroxyindole acetic acid in brain was found in human CYP2D6-transgenic mice than wild-type mice and the transgenic mice were more able to adapt to anxiety (Cheng et al., 2013), providing direct evidence that brain CYP2D6 catalyzes 5-HT formation from 5-MT (Figure 1B). 5-MT is formed via the deacetylation of melatonin both in brain and liver (Beck and Jonsson, 1981; Hardeland, 2010). 5-HT is mainly involved in some basic behavior and emotion regulation, and lack of 5-HT may lead to depressive disorder. Mouse experiments showed the decreased activity of cerebellar CYP2D by quinine resulted in a behavioral deficit related to spatial learning and memory, as well as increased anxiety-related behavior (Zhang et al., 2018), further demonstrating roles of brain CYP2D-mediated 5-HT formation. Melatonin was also detected (via its deacetylation to 5-MT) to support 5-HT synthesis via CYP2D in brain (Haduch et al., 2016). Thereby, we hypothesize that elevation of brain CYP2D under depressive disorder (Haduch et al., 2018) may increase melatonin consumption, causing sleep disorders in patients with depressive disorder.

5-HT is mainly metabolized by MAO and aldehyde dehydrogenase to 5-hydroxyindole acetic acid. 5-HT glucuronide was detected in microdialysis samples of rat brain, and its concentration was 2.5 times higher than that of 5-HT (Uutela et al., 2009). The glucuronide conjugate of 5-HT was also detected in human brain microdialysis samples (Suominen et al., 2013), suggesting that glucuronidation in human brain may be an important phase Ⅱ metabolism pathway of 5-HT (Figure 1B). 5-HT is the specific probe substrate of UGT1A6. The mRNA expression of *Ugt1a6* was detected in brain of rats (Sakakibara et al., 2016). In humanized *UGT1* mice, brain *UGT1A6* was also found and the brain microsomes exhibited glucuronidation activity toward 5-HT (Kutsuno et al., 2015), demonstrating an important role of UGT1A6 in brain 5-HT elimination.

### Bilirubin

Bilirubin, which is poorly water-soluble, exists in the form of its glucuronide conjugate under normal conditions. Bilirubin glucuronidation is mainly mediated by UGT1A1, which is the rate-limiting step for bilirubin bile excretion and detoxification (Wang et al., 2016). Neonatal hyperbilirubinemia mostly results from the delayed UGT expression in liver (Rets et al., 2019; Nguyen et al., 2020). Bilirubin is an important endogenous antioxidant that protects neurons from free radicals. However, bilirubin glucuronidation deficiency in liver leads to excessive free bilirubin, which can enter the brain and impair CNS. In human brain, *UGT1A1* mRNA was detected (Kutsuno et al., 2015), but bilirubin glucuronidation was not (Ouzzine et al., 2014). A report showed that UGT1A1 might not be the main isoform catalyzing the glucuronidation in rat brain (Asai et al., 2017). Several reports indicated that brain mitochondria and microsomal CYPs also metabolized bilirubin (Hansen and Allen, 1997; Hansen, 2000). In hyperbilirubinemic Gunn rat pups with congenital deficiency of hepatic UGT1A1, intraperitoneal sulfadimethoxine reduced brain bilirubin concentration, and the extent of decrease in bilirubin accumulation was closely related with the induction of *Cyps* (mainly *Cyp1a1*, *Cyp1a2* and *Cyp2a3*) mRNA in different brain regions (Gazzin et al., 2012). Data from primary rat astrocytes of cortex and cerebellum showed that β-naphthoflavone (an inducer of CYP1A1 and CYP1A2) enhanced bilirubin oxidation and improved the cell viability, in line with induction of CYP1A1 and CYP1A2 (Gambaro et al., 2016). However, a recent report showed that under physiological conditions, brain microsomal CYP1A2 was incapable of metabolizing bilirubin without inducers (Li et al., 2019). Thus, the roles of brain CYP1A2 in bilirubin metabolism need further investigation. Additionally, aromatic hydrocarbon receptor (AHR) regulates the expressions of various enzymes and transporters, including CYP1A1 and CYP1A2 (Qin et al., 2019). Bilirubin, as an agonist of AHR, itself may trigger its metabolism in brain via inducing CYP1A2.

### Arachidonic Acid

Arachidonic acid (AA), the most abundant omega-6 polyunsaturated 20-carbon fatty acid (PUFA) in brain, is derived from linoleic acid. AA is primarily metabolized by cyclooxygenase (COX), lipoxygenase (LOX), and CYPs to prostaglandins, thromboxanes, leukotrienes and other eicosanoid derivatives, which are essential for the human cardiovascular and immunity system.

CYP epoxygenases mediate the oxidation of AA to epoxyeicosatrienoic acids (EETs), which also occur in the human brain. CYP2C8/9 and CYP2J2 are the main epoxygenases in human tissues, including endothelial cells where EETs are mainly generated (Shahabi et al., 2014). EETs are released from astrocytes, neurons and endothelial cells, acting as powerful vasodilators to increase blood flow (Carver et al., 2014), or anti-inflammatory agents to extensively protect from cerebrovascular diseases. Inflammation may decrease expressions and activity of CYP epoxygenases in heart, renal, liver and brain. In the rat primary astrocytes, it was found that the inflammatory response induced by lipopolysaccharide down-regulated the expressions and functions of CYP2J3 and CYP2C11, which was reversed by selective NF-κB inhibitor IMD-0354 (Navarro-Mabarak et al., 2019). Inflammation inhibits EETs formation via down-regulating the enzymes, leading to enhancement of inflammatory response, further suppressing the enzymes and EETs production, forming a vicious circle in brain. CYP epoxygenases may serve as the therapeutic targets of chronic cerebrovascular diseases and brain inflammatory disorders via blocking the vicious circle. EETs also inhibit oxidative stress and neuroinflammation to act its neuroprotective role, indicating that it has the potential to treat PD (Lakkappa et al., 2016; Lakkappa et al., 2018; Huang et al., 2018). AA is also converted to anandamide, which can be further metabolized by brain CYP3A4, CYP4F2 and CYP2D6 (Snider et al., 2010). Anandamide and its metabolites have different ability and mechanism of anti-inflammation (McDougle et al., 2017).

CYP1B1 (Choudhary et al., 2004) and CYP2U1 (Devos et al., 2010) were reported to preferentially catalyze AA to hydroxyeicosatetraenoic acids (HETEs) in brain. Moreover, in freshly isolated human brain microvasculature, only CYP1B1 and CYP2U1 could be quantified among the investigated 21 CYPs (Shawahna et al., 2011). Glutamate also induced the expressions and functions of CYP1B1 and CYP2U1 in astrocytes via mGlu5 receptor, suggesting that the neuron-astrocyte reciprocal signaling may affect the CYPs-mediated metabolism of AA (Yu et al., 2019).

Overall, under pathophysiologic conditions, some CYPs affect the blood flow in the brain environment, the infant growth and brain development (Hadley et al., 2016), immunity and immune response and inflammation via altering metabolism of AA.

### Cholesterol

Cholesterol not only participates in the formation of cell membranes, but also is a raw material for the synthesis of bile acids, vitamin D and steroid hormones. About 25% of the body’s cholesterol is concentrated in brain. Cholesterol plays a very important role in brain growth. Since cholesterol can hardly pass BBB, most of the brain cholesterol is synthesized in brain rather than obtained from plasma. Brain CYPs are essential for the biosynthesis and excretion of cholesterol.

CYP51 is the only CYP participating in cholesterol biosynthesis by removal of the 14α-methyl group of lanosterol, and it is ubiquitously expressed, including brain microsomes (Aoyama et al., 1996). Cholesterol 24-hydroxylase is a type of CYPs (CYP46A1), which is selectively expressed in brain, including hippocampus, cortex and cerebellum (Lund et al., 1999). CYP46A1 is mainly involved in the catabolism of most cholesterol, and generated 24-S- hydroxycholesterol diffuses across BBB easily. It is the dominating pathway for cholesterol elimination from brain (Björkhem et al., 1998). Interestingly, CYP46A1 seems to regulate new cholesterol synthesis in brain. In *Cyp46a1* ^−/−^ mice, the synthesis of new cholesterol in brain was reduced by about 40% of wild type mice (Lund et al., 2003), demonstrating a crucial role of CYP46A1 in brain cholesterol turnover. In addition, CYP11A1, CYP27A1 and CYP7A1 metabolize cholesterol to pregnenolone, 27-hydroxycholesterol and 7α-hydroxycholesterol in brain, respectively (Dutheil et al., 2008).

Several studies have demonstrated that the levels of brain cholesterol and its metabolites are closely related to Alzheimer’s disease (AD) (Hughes et al., 2013; Djelti et al., 2015). Moreover, AD also influence CYP46A1 expression and function (Burlot et al., 2015), in turn, alter cholesterol metabolism. In addition, cholesterol homeostasis in brain is also involved in Huntington’s disease and Parkinson’s disease (PD) (Björkhem et al., 2013; Boussicault et al., 2016; Nóbrega et al., 2020).

### Allopregnanolone

Allopregnanolone is mainly synthesized from progesterone in brain corticolimbic glutamatergic neurons (Pinna, 2019). The 5α-reductase enzyme first converts progesterone to 5α-dihydroprogesterone, which is further metabolized by 3α-hydroxysteroid dehydrogenase to form allopregnanolone. Allopregnanolone, acting as a potent endogenous positive allosteric modulator of gamma-aminobutyric acid (GABA) A receptor, regulates emotional behaviors, reducing brain excitability and eliciting sedative-hypnotic, anxiolytic, and anticonvulsant effects (Yilmaz et al., 2019; Lloyd-Evans and Waller-Evans, 2020). Allopregnanolone has been approved for treatment of postpartum depression (Zorumski et al., 2019). 21-Hydroxylation of allopregnanolone is catalyzed by brain CYP2D (Kishimoto et al., 2004). Some antidepressants such as fluoxetine inhibited CYP2D-mediated 21-hydroxylation of allopregnanolone (Niwa et al., 2008), indicating that it may affect neurosteroid metabolism, further leading to the increase of allopregnanolone concentration in the brain (Uzunov et al., 1996; Pinna et al., 2006), which in turn may modify the pharmacological action of antidepressants. Clinical studies have showed the level of allopregnanolone was significantly lower in the CSF of depressed patients and could be normalized by treatment with fluoxetine and fluvoxamine (Romeo et al., 1998; Uzunova et al., 1998), in which brain CYP2D may have certain roles.

## Alterations in Expressions and Functions of Cytochrome P450s and UDP-Glucuronosyltransferases Under Diseases and Their Impacts on Drug Disposition

Accumulating evidence has demonstrated that some diseases, especially CNS diseases, are often accompanied by changes of CYPs and UGTs in brain. The alterations in expressions and functions of brain CYPs and UGTs by diseases lead to disorders of above-mentioned endogenous substances in brain or affect the efficacy of therapeutic drugs, in turn, aggravating the disease progression.

### Epilepsy and Drug Resistance

Epilepsy is a common chronic CNS disease, in which the sudden abnormal discharge of brain neurons leads to transient brain dysfunction. Several investigations have demonstrated that epilepsy altered expressions and functions of drug transporters (Luna-Tortós et al., 2010; Ma et al., 2013; Sun et al., 2016) and enzymes (Ghosh et al., 2010; Williams et al., 2019) in BBB and brain, which affects the bioavailability of antiepileptics through BBB, leading to pharmacoresistance.

Most of CYP3A4 in brains are mainly distributed in neurons (Ghosh et al., 2011), which may be affected by epilepsy. The report showed that the level of CYP3A4 protein in brain was positively correlated with carbamazepine metabolism (Ghosh et al., 2011), suggesting that CYP3A4 exhibits certain activity in brain. Overexpression of CYP3A4 protein was found in primary endothelial cells established from brain specimens from drug-resistant epileptic patients (Ghosh et al., 2010). Data on brain specimens surgically resected from patients with epilepsy also showed that the protein level and activity of CYP3A4 in the brain region of epilepsy lesions were significantly increased compared to non-epileptic regions (Williams et al., 2019). A report explained that overexpression of CYP3A4 had close correlation with pregnane X and glucocorticoid receptors in endothelial cells from epileptic patients (Ghosh et al., 2017). Fluorescent immunostaining and Western blot showed that epilepsy increased protein levels of both ABC transporters and CYPs in BBB (Ghosh et al., 2011). The increased ABC transporters and CYPs synergistically decreased the bioavailability of drugs through BBB, aggravating drug resistance. Notably, increased CYP3A4 in BBB metabolizes some antiepileptics to neurotoxic metabolites, also worsening epilepsy. Data on *in vitro* BBB established with endothelial cells from epileptic patients confirmed that carbamazepine was metabolized to quinolinic acid (Ghosh et al., 2012), a seizure agonist which leads to neurotoxicity. Quinolinic acid was also detected in brain of epileptic patients receiving carbamazepine, but not in epileptic patients receiving other antiepileptics or control patients (Ghosh et al., 2012). In addition, administration of antiepileptics metabolized by CYPs increased CYP3A4 protein level in both epileptic and non-epileptic brain regions of epileptic patients, then enhancing metabolism of drugs in brain and aggravating drug resistance (Williams et al., 2019). Overall, abnormal elevation of CYP3A4 under epilepsy has pathological relevance to the mechanism of drug-resistance epilepsy. Meanwhile, CYP3A4 can mediate the metabolism of some CNS drugs, so we need take its alteration into account to guide clinical medication.

CYP2D, expressed in various human brain regions, also metabolizes a variety of CNS drugs. It was found that status epilepticus down-regulated the mRNA expression and activity of CYP2D4 in the cortex and hippocampus of rats (Asai et al., 2018b). Severe status epilepticus increased CYP2E1 protein level in the hippocampus of mice, which was localized and cell specific, and antiepileptics phenytoin further induced localized CYP2E1 protein level in brain (Boussadia et al., 2014). Spontaneous recurrent seizure also up-regulated CYP3A13 and CYP2E1 mRNA and protein expressions in the liver and the hippocampus of mice, perhaps related to nuclear receptors or inflammatory pathways (Runtz et al., 2018). The additional investigation is needed to clarify the importance of alterations in CYP2D and CYP2E1 under epilepsy.

Status epilepticus significantly up-regulated expressions of *Ugt1a1* and *Ugt1a7* mRNA in cortex and hippocampus of rat but not in liver. The up-regulation of *Ugt1a1* and *Ugt1a7* in brain was considered to be related to oxidative stress (Asai et al., 2018a). UGT1A4 catalyzes the N-glucuronidation of some antiepileptics (such as lamotrigine and midazolam), tricyclic antidepressants (such as doxepin, imipramine and amitriptyline) and antipsychotics (such as clozapine, olanzapine and trifluoperazine). Cerebral microvascular endothelial cells from epileptic patients receiving lamotrigine displayed high protein expression and activity of UGT1A4, accompanied by existence of lamotrigine 2-N glucuronide (Ghosh et al., 2013). All these results indicate that the overexpression of UGTs may also enhance pharmacoresistance to antiepileptics.

### Parkinson’s Disease

Parkinson’s disease (PD) is a common neurodegenerative disease. Its main pathological change is the degeneration and death of dopaminergic neurons in the substantia nigra of the midbrain, which causes a significant decrease in the DA content of the striatum and then leads to disease.

As we mentioned above, brain CYP2D also participates in the synthesis of DA and the elimination and metabolism of neurotoxic substances (Stocco et al., 2020), so the decrease in CYP2D has the potential to induce PD. Noteworthy, CYP2D6 protein level in human brain increased with age but decreased approximately 40% in several brain regions of PD cases (Mann et al., 2012). The mice with PD induced by 1-methyl-4-phenyl-1,2,3,6-tetrahydropyridine (MPTP) or pesticides like maneb and qaraquat, had the decreased mRNA expression of *Cyp2d22* (homolog of human CYP2D6) in nigrostriatal tissues, while nicotine or resveratrol showed the neuroprotection via up-regulating *Cyp2d22* expression (Singh et al., 2009; Srivastava et al., 2012), demonstrating the neuroprotective effects of CYP2D22/CYP2D6-mediated DA synthesis or neurotoxin metabolism (Fernandez-Abascal et al., 2018).

CYP2E1 is detected in substantia nigra, the pathophysiology-related brain regions of PD. There was an certain association between the intron 7 polymorphism of *CYP2E1* gene and PD in a Swedish population (Shahabi et al., 2009). *CYP2E1* gene was hypomethylated and overexpressed in PD patients’ brains (Kaut et al., 2012). In addition, it has also been reported that CYP2E1 in brain can mediate the generation of reactive oxygen species (ROS) (Doycheva et al., 2019; Moyano et al., 2019) and regulate DA release, which are closely related to the pathogenesis of PD (Shahabi et al., 2008).

### Alzheimer’s Disease

Alzheimer’s disease (AD) is a common neurodegenerative disease characterized by progressive cognitive dysfunction and memory impairment. It is generally believed that AD is the result of both genetic and environmental factors. There are many hypotheses about its pathophysiological mechanism, including central cholinergic nerve damage, amyloid β (Aβ) protein toxicity, Tau protein abnormal phosphorylation, free radical damage, etc.

Cholesterol increases the production and deposition of Aβ peptides, leading to the formation of amyloid plaques, which is a pathological feature of AD. Cholesterol in brain is mainly metabolized into 24-S-hydroxycholesterol through CYP46A1, an enzyme expressed only in neurons. Compared with control mice, the amounts of CYP46A1 protein and 24-S-hydroxycholesterol in the hippocampus was lower in THY-Tau22 mice, a model of AD-like Tau pathology without amyloid pathology (Burlot et al., 2015), indicating that AD may decrease the expression and activity of CYP46A1. And then injection of adeno-associated vector (AAV) encoding *CYP46A1* into the hippocampus of THY-Tau22 mice led to CYP46A1 content normalization and completely rescued memory impairment (Burlot et al., 2015). In line, AAV-mediated neuronal *CYP46A1* overexpression in AD mouse models significantly reduced occurrence of amyloid plaques (Hudry et al., 2010). In addition, Aβ significantly reduced CYP epoxygenases activities in specific regions and cells, decreasing levels of EETs and aggravating AD (Sarkar et al., 2011). These results demonstrate that the down-regulation of brain CYP46A1 or CYP epoxygenases by AD leads to the disorders of cholesterol or EETs, in turn, exacerbating symptoms in AD. Recent studies found the anti-HIV drug efavirenz activated CYP46A1 at the low dose, which could be a new anti-AD treatment (Mast et al., 2017; Mast et al., 2020; van Lier, 2020).

### Depressive Disorder and Psychotropic Medications

Depressive disorder is a type of mental illness, whose common symptoms include low spirits, emotional indifference, sleep disorders and slow thought. 5-HT deficiency in brain is one of its pathogeneses. Accumulating evidence has demonstrated that brain CYP2D and UGT1A6 participate in the synthesis and metabolism of 5-HT, and CYP2D has relevance to depressive disorder (Ingelman-Sundberg et al., 2014; Peñas-Lledó et al., 2015).

The alterations in metabolic enzymes by depressive disorder and psychotropic medications in brain are often different from those in liver. *In vitro* microsomal data showed that chronic mild stress depression increased CYP2D activity in the hippocampus of rats but not in liver. In stressed rats treated with escitalopram or venlafaxine, it was found that activity of CYP2D was increased in brain but decreased in liver (Haduch et al., 2018). Other antidepressants such as paroxetine inhibited brain CYP2D activity (Niwa and Sugimoto, 2019). *In vivo* data showed that effects of psychotropic drugs on rat brain CYP2D were dependent on brain regions. Fluoxetine decreased CYP2D protein expression and activity in the striatum and nucleus accumbens, but elevated CYP2D protein expression in the cerebellum. Thioridazine decreased CYP2D activity in the substantia nigra and nucleus accumbens, but significantly increased that activity in the striatum and cerebellum. Clozapine significantly enhanced CYP2D activity in the truncus cerebri (Haduch et al., 2011). Additionally, mirtazapine and imipramine affected expression and activity of CYP2D in liver (Daniel et al., 2002; Haduch et al., 2020a), but not in brain (Haduch et al., 2011). The effects of psychotropic drugs on brain CYP2D expression and activity are complicated, but all these results indicate brain CYP2D is susceptible. More importantly, brain CYP2D mediates synthesis or metabolism of endogenous substances (DA, 5-HT and allopregnanolone) and exogenous compounds, which may affect disease progression, so we need to take the alterations of CYP2D into account during treatment with psychotropic drugs. Additionally, CYP46A1-mediated cholesterol metabolism had certain relevance to depressive disorder (Popiolek et al., 2020; Na et al., 2021).

Brain CYP2D also metabolizes a variety of antipsychotics, affecting the levels of drugs and their metabolites in brain, in turn, altering drug response. Some CYP2D-mediated metabolites of antipsychotics have potential neurotoxicity. Moreover, their side effects and drug efficacy are not necessarily related to the concentration of drugs in plasma. For example, CYP2D-mediated metabolites of haloperidol in brain may induce catalepsy, while haloperidol may induce vacuous chewing movements. Rat experiments showed that intracerebral administration of propranolol selectively inhibited activity of brain CYP2D, leading to increases in vacuous chewing movements after chronic haloperidol, while subcutaneous nicotine pre-treatment increased catalepsy after acute haloperidol due to induction of brain CYP2D (Miksys et al., 2017), which was also confirmed in humanized CYP2D6 transgenic mice (Tolledo et al., 2020). However, liver CYP2D activity and plasma haloperidol concentration were unaltered by propranolol and nicotine (Miksys et al., 2017), which highlights the importance of brain CYP2D. CYP2D6 polymorphism also affects drug response after antipsychotic treatment. In a clinic trial from Korea, according to brain magnetic resonance imaging and genotyping for CYP2D6, all participants with schizophrenia were classified as intermediate metabolizers and extensive metabolizers. Following 4 weeks of antipsychotic treatment, compared with the intermediate metabolizers, the extensive metabolizers showed more improvements in positive symptoms which were negatively associated with fractional anisotropy values, and positively associated with radial diffusivity values in the right hippocampal region. Both values were also significantly correlated with CYP2D6 activity (Shin et al., 2021). Thereby, decreased CYP2D6 activity may confer susceptibility to the development of schizophrenia by increasing hippocampal hyperactivity and dopaminergic burden. It was also confirmed that CYP2D6 variations contributed to schizophrenia risk (Ma et al., 2020).

### Smoking and Alcoholism

CYPs in brain are easily induced by addictive substances such as ethanol and nicotine. Ethanol and nicotine were reported to induce CYP2E1 protein expression in both liver and brain of rats (Howard et al., 2003; Yue et al., 2009). The elevated brain CYP2E1 by ethanol led to overmuch ROS or the loss of synaptic proteins via down-regulating peroxisome proliferator-activated receptor alpha (PPARα), contributing to ethanol-induced neurotoxicity (Zhong et al., 2012; Na et al., 2017). CYP2D protein levels in monkey brain but not liver, was induced by nicotine and ethanol (Miller et al., 2014). Smokers often have higher levels of brain CYP2D6, especially in the basal ganglia (Mann et al., 2008). Compared with normal people, smokers have a lower risk of suffering from PD (Lee et al., 2018; Mappin-Kasirer et al., 2020), indicating that CYP2D6 may have a certain neuroprotective effect. In addition, brain CYP2D metabolizes many addictive drugs, including methamphetamine, consequently to alter their responses and addictions (Stocco et al., 2021), particularly under induction of brain CYP2D. Human CYP2B6 and rat homolog CYP2B1 share 75% of amino acid properties, have overlapping substrate specificities, and metabolize a variety of substrates, including nicotine (Bloom et al., 2019), drugs of abuse, neurotoxins, anticancer drugs, anesthetics and 5-HT. CYP2B is expressed differently in various regions of rat brain and could be induced by nicotine, but not in liver, which lowered brain nicotine levels independent of liver, increasing withdrawal symptoms (Garcia et al., 2015, 2017). Ethanol could induce CYP2B1/2 mRNA, protein and activity in rat liver rather than brain (Schoedel et al., 2001) while in the caudate, putamen and cerebellum of African green monkeys, CYP2B6 protein was induced by ethanol (Ferguson et al., 2013), which also affected nicotine oxidation in the body. Considering these enzymes in brain contributes to understanding cross-tolerance and co-abuse of ethanol and nicotine.

Nicotine is also metabolized by UGTs (Murphy et al., 2014), which may affect the addictiveness of nicotine. Brain *UGT1A3* mRNA was induced by nicotine in humanized *UGT1* mice and UGT1A3 metabolizes various exogenous and endogenous compounds, such as antidepressants (amitriptyline) and chenodeoxycholic acid (Sakamoto et al., 2015). Several studies have shown that multiple UGTs such as UGT1A1, UGT1A6 and UGT2B7 mediate glucuronidation of ethanol (Foti and Fisher, 2005; Schwab and Skopp, 2014). High concentration of ethyl glucuronide was detected in CSF and brain of patients who died of acute alcoholism (Kugelberg and Jones, 2007), demonstrating roles of brain UGTs in metabolism of ethanol.

### Morphine and Other Central Nervous System Analgesics Disposition

**FIGURE 2:**
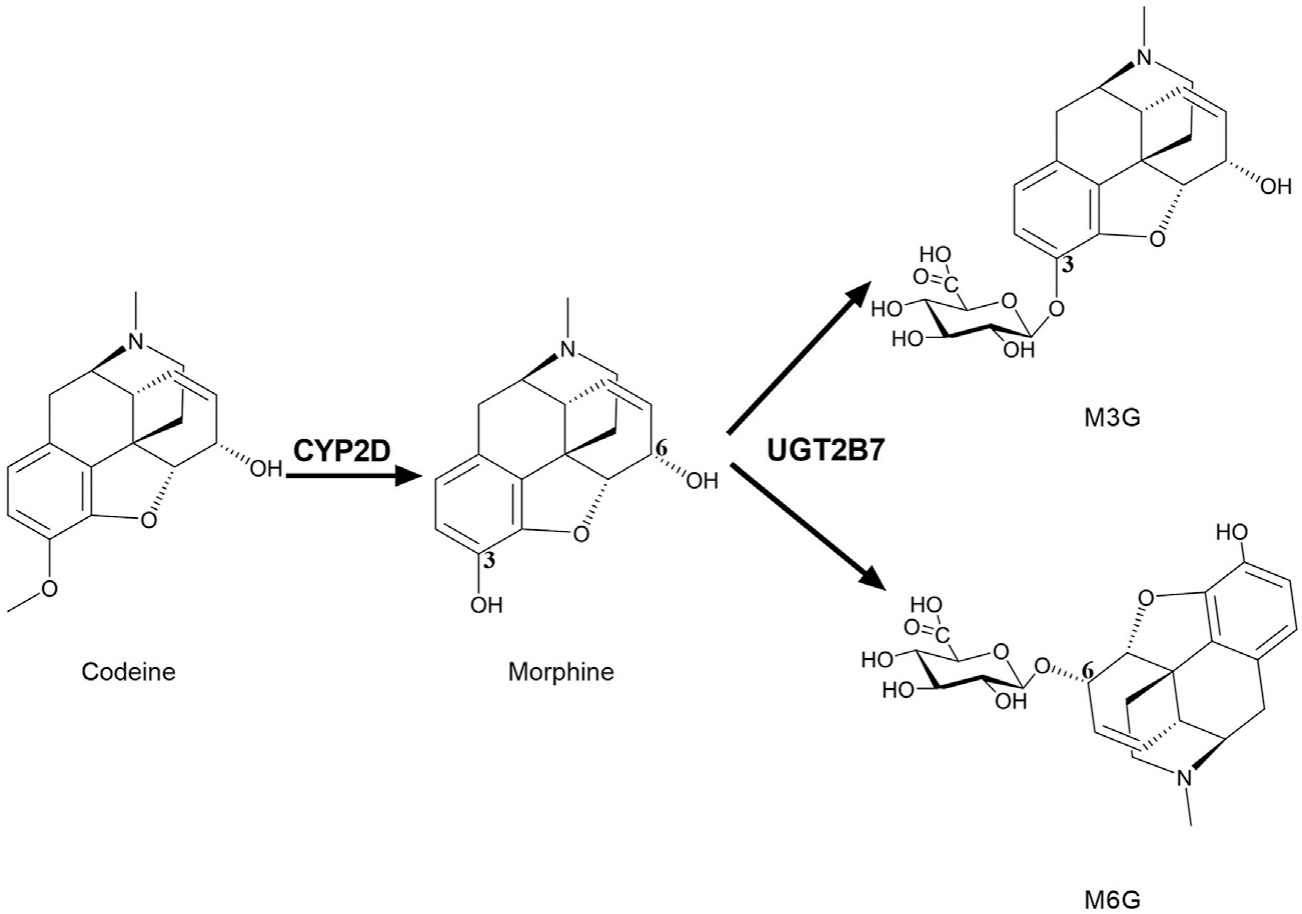
Brain CYP2D and UGT2B7 mediate the metabolism of codeine and morphine. CYP2D catalyzes the demethylation of codeine into morphine. UGT2B7 mediates the glucuronidation of positions 3 and 6 of the hydroxyl groups of morphine into M3G and M6G. CYP2D, Cytochrome P450 2D; UGT2B7, UDP-glucuronosyltransferase 2B7; M3G, morphine-3-glucuronide; M6G, morphine-6-glucuronide.

Many opioids are metabolized by CYPs, and their metabolites have medicinal activity (Smith, 2011). There are always great individual differences in the efficacy of drugs acting on CNS, which may be closely related to the differences in brain drug metabolic enzymes. CYP2D metabolizes codeine into the active metabolite morphine to enhance its analgesic effect, since the affinity of morphine to opioid receptors in the rat brain is 3,000 times stronger than that of codeine (Pert and Snyder, 1973). Compared with codeine, morphine is more difficult to pass BBB and exhibits active efflux in BBB (Oldendorf et al., 1972), thus, the analgesic effect of codeine mainly depends on its conversion to morphine by brain CYP2D (Figure 2). It was found that intracerebroventricular injections of CYP2D inhibitors (propranolol or propafenone) significantly decreased the concentration of morphine in rat brain and weakened the analgesic activity of codeine after peripheral administration of codeine (Zhou et al., 2013). On the contrast, nicotine increased the analgesic effect of codeine via inducing brain CYP2D (McMillan and Tyndale, 2015). Furthermore, the elevation of brain CYP2D increased the rate of codeine tolerance (McMillan and Tyndale, 2017). Brain CYP2D-mediated transformation from some other opioids (tramadol and oxycodone) to their metabolites (O-desmethyltramadol and oxymorphone) also affects their analgesia (Wang et al., 2015; McMillan et al., 2019, 2020). It is worth noting that characteristics of drug-drug interactions in brain are different from liver. It was found that testosterone inhibited the catalytic function of CYP2D in SH-SY5Y and U251 cells but not in HepG2. *In vivo* data showed that testosterone down-regulated protein expression of CYP2D in brain but not liver of rats. In line, administration of testosterone decreased the active metabolite O-desmethyltramadol of tramadol in CSF rather than in plasma to weaken analgesia of tramadol. On the contrast, orchiectomy increased protein expression of CYP2D in brain but not liver, and then elevated O-desmethyltramadol levels in CSF but not plasma to enhance analgesia of tramadol, which were attenuated by supplement of testosterone (s. c) or propranolol (i. c. v) (Li et al., 2015).

Similarly, in human brain, UGT2B7 is considered to mainly mediate morphine glucuronidation into two metabolites, morphine-3-glucuronide (M3G) and morphine-6-glucuronide (M6G) (Figure 2) (Yamada et al., 2003; Abildskov et al., 2010; Yueh et al., 2011). M3G and M6G were also detected in primary microglia of neonatal rat treated with morphine (Togna et al., 2013). M3G is analgesically inactive, while M6G is active (Nagano et al., 2000; De Gregori et al., 2012). M6G passes BBB more slowly than morphine (Bouw et al., 2001) and shows stronger analgesic effects, so the alterations in UGTs may affect analgesia of morphine. Thus, the brain CYPs and UGTs are particularly important for the exposure of analgesic drugs such as morphine in brain.

## Conclusions and Perspectives

**FIGURE 3:**
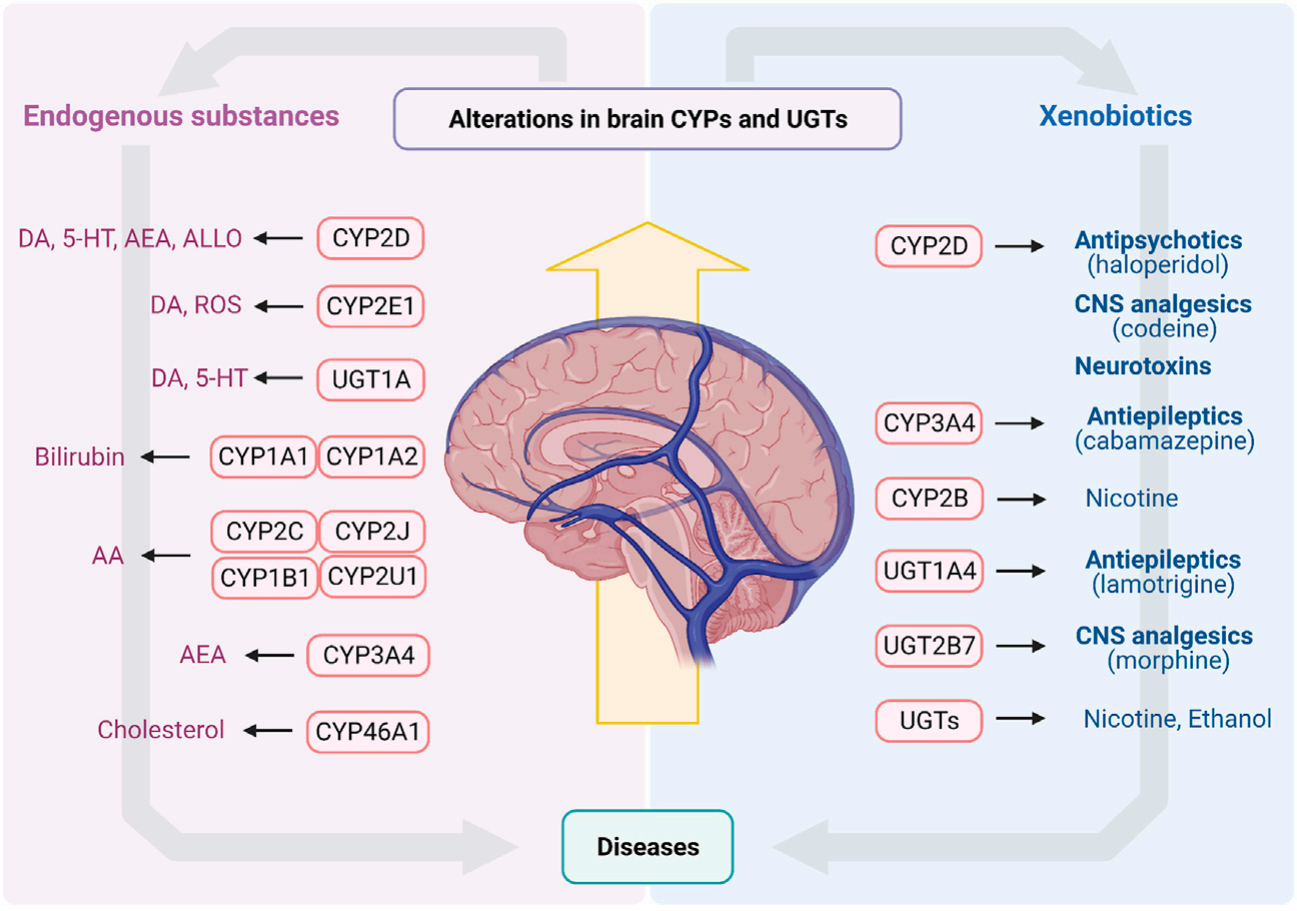
The endogenous substances and xenobiotics metabolized by CYPs and UGTs in brain. AA, arachidonic acid; AEA, arachidonoyl ethanolamine (anandamide); ALLO, allopregnanolone; CNS, central nervous system; CYPs, Cytochrome P450s; DA, dopamine; ROS, reactive oxygen species; UGTs, UDP-glucuronosyltransferases; 5-HT, serotonin.

Brain CYPs and UGTs play a significant role in metabolism of endogenous substances and CNS drugs (Figure 3). *In vitro* and *in vivo* studies confirm that these enzymes mediate catabolism/anabolism of endogenous substances (such as DA, 5-HT, bilirubin, AA, cholesterol and allopregnanolone) to maintain brain homeostasis. Furthermore, for some CNS drugs disposition, CYPs and UGTs in brain are of greater importance than those in liver, such as CYP2D, CYP3A4, UGT1A4 and UGT2B7. More importantly, brain CYPs and UGTs are susceptible to some diseases and drugs, while the alterations of them may lead to the disorders of endogenous substances or the change of drug disposition, aggravating disease progression. The overexpression of brain CYP3A4 or UGT1A4 under epilepsy may cause drug resistance. PD significantly suppresses brain CYP2D, in turn, decreasing DA content or increasing neurotoxic substances, to result in PD exacerbation. Some psychotropic medications also affect the expression and activity of brain CYP2D, which may affect levels of DA, 5-HT and allopregnanolone or induce drug-drug interaction to alter therapeutic efficiencies. Nicotine and alcohol may also have an impact on multifarious enzymes in brain, altering disease progression or drug disposition.

Some diseases alter brain CYPs and UGTs (Table 1), further affecting the levels of endogenous substances or drugs metabolized by these enzymes. Clarifying the correlation between them and the significance of these enzymes on disease progression and drug efficacy may eventually provide guidance on medication under diseases, explain the accumulation or resistance of some CNS drugs and set new therapeutic targets for some CNS diseases.

**TABLE 1:** The alterations in CYPs and UGTs under diseases.

Metabolic enzymes	Diseases	Alteration	Brain regions	References
CYP2D	Status epilepticus	mRNA and activity ↓	Cortex, hippocampus	Asai et al. (2018b)
	PD	mRNA and activity ↓	Nigrostriatal tissues	Singh et al. (2009), Srivastava et al. (2012)
	Smoking	Protein and activity ↑	Especially basal ganglia	Mann et al. (2008), McMillan and Tyndale (2015)
	Alcoholism	Protein ↑	Most brain regions	Miller et al. (2014)
	Depressive disorder	Activity ↑	Hippocampus	Haduch et al. (2018)
CYP3A4	Epilepsy	Protein and activity ↑	Epilepsy lesions, BBB	Ghosh et al. (2010), Williams et al. (2019)
CYP2E1	Status epilepticus	Protein ↑	Hippocampus	Boussadia et al. (2014)
	PD	mRNA ↑	Cortex	Kaut et al. (2012)
	Smoking	Protein ↑	Frontal cortex, putamen	Ferguson et al. (2013)
	Alcoholism	Protein and activity ↑	Hippocampus, cerebellum, brainstem	Zhong et al. (2012)
CYP2B	Smoking	mRNA, protein and activity ↑	Brain stem, frontal cortex, striatum, olfactory tubercle	Miksys et al. (2000), Garcia et al. (2017)
	Alcoholism	Protein ↑	Caudate, putamen, cerebellum	Ferguson et al. (2013)
CYP2C, CYP2J	Inflammation	mRNA, protein and activity ↓	—	Navarro-Mabarak et al. (2019)
CYP46A1	AD	Protein and activity ↓	Hippocampus	Burlot et al. (2015)
UGT1A4	Epilepsy	Protein and activity ↑	BBB	Ghosh et al. (2013)
UGT1A1, UGT1A7	Status epilepticus	mRNA ↑	Cortex, hippocampus	Asai et al. (2018a)
UGT1A3	Smoking	mRNA ↑	—	Sakamoto et al. (2015)

AD, alzheimer’s disease; BBB, blood-brain-barrier; CYPs, Cytochrome P450s; PD, Parkinson’s disease; UGTs, UDP-glucuronosyltransferases.

## Abbreviations

AA, arachidonic acid; AAV, adeno-associated vector; AD, Alzheimer’s disease; AHR, aromatic hydrocarbon receptor; Aβ, amyloid β; BBB, blood-brain-barrier; CNS, central nervous system; COMT, catechol methyltransferase; COX, cyclooxygenase; CSF, cerebrospinal fluid; CYPs, Cytochrome P450s; DA, dopamine; DOPAC, dihydroxyphenyl acetic acid; EETs, epoxyeicosatrienoic acids; GABA, gamma-aminobutyric acid; HETEs, hydroxyeicosatetraenoic acids; HVA, homovanillic acid; l-dopa, levodopa; LOX, lipoxygenase; MAO, monoamine oxidase; MPTP, 1-methyl-4-phenyl-1,2,3,6-tetrahydropyridine; M3G, morphine-3-glucuronide; M6G, morphine-6-glucuronide; PD, Parkinson’s disease; PPARα, peroxisome proliferator-activated receptor alpha; PUFA, polyunsaturated fatty acid; ROS, reactive oxygen species; UGTs, UDP-glucuronosyltransferases; 5-HT, serotonin; 5-MT, 5-methoxytryptamine.
